# Testing for nested oscillation

**DOI:** 10.1016/j.jneumeth.2008.06.035

**Published:** 2008-09-15

**Authors:** W.D. Penny, E. Duzel, K.J. Miller, J.G. Ojemann

**Affiliations:** a Wellcome Trust Centre for Neuroimaging, University College, London WC1N 3BG, UK; b Institute for Cognitive Neuroscience, University College, London WC1N 3BG, UK; c Department of Physics, University of Washington, Box 351560, Seattle, WA 98195-1560, USA; d Neurological Surgery, University of Washington, Box 351560, Seattle, WA 98195-1560, USA

**Keywords:** Intracranial EEG, Phase coupling, Cross-frequency coupling, Oscillations

## Abstract

Nested oscillation occurs when the amplitude of a faster rhythm is coupled to the phase of a slower rhythm. It has been proposed to underlie the discrete nature of perception and the capacity of working memory and is a phenomenon observable in human brain imaging data. This paper compares three published methods for detecting nested oscillation and a fourth method proposed in this paper. These are: (i) the modulation index, (ii) the phase-locking value (PLV), (iii) the envelope-to-signal correlation (ESC) and (iv) a general linear model (GLM) measure derived from ESC. We applied the methods to electrocorticographic (ECoG) data recorded during a working-memory task and to data from a simulated hippocampal interneuron network. Further simulations were then made to address the dependence of each measure on signal to noise level, coupling phase, epoch length, sample rate, signal nonstationarity, and multi-phasic coupling. Our overall conclusion is that the GLM measure is the best all-round approach for detecting nested oscillation.

## 1 Introduction

Nested oscillation, otherwise referred to as phase-amplitude coupling (PAC), occurs when the amplitude of a faster rhythm is coupled to the phase of a slower rhythm. This phenomenon has received an increasing amount of attention in recent years, (Buzsaki, 2006; Jensen and Colgin, 2007; Cohen, 2008) and has been proposed as a mechanism for the capacity of working memory, the discrete nature of perception and plays a role in sleep (Steriade, 2006) and olfaction (Kepecs et al., 2006).

Lisman and Idiart (1995) have suggested that nested theta and gamma oscillations underlie the specific capacity limits of working memory (WM). The approximately seven gamma cycles that can fit into a theta cycle are proposed to correspond to the seven plus or minus two items that can be stored in working memory. See Jensen (2006) for a recent discussion of the computational models and physiological evidence supporting this idea.

In a recent review, VanRullen and Koch (2003) postulated that alpha and nested gamma cycles produce ‘discrete perception’ in which gamma waves contain the information of each snapshot, with the organization of the ensemble of snapshots mediated by the alpha waves. That is, those events represented within the same alpha cycle form part of the same percept. The finding that approximately four objects can be perceived at a glance is proposed to be a result of such alpha–gamma nesting. See Palva and Palva (2007) for a recent review.

Reports of nested oscillations are also now emerging from human brain imaging studies. Vanhatalo et al. (2004) describe ‘infra-slow oscillations’ (ISOs) in human EEG during sleep at frequencies between 0.02 and 0.2 Hz. These ISOs modulate cortical excitability such that increases in power above 1 Hz occur at ISO troughs. Also, the frequency of discrete EEG events such as K-complexes and intra-ictal epileptic spikes are increased at the troughs. In addition to their primary findings, Vanhatalo et al. (2004) also found increase in theta (4–8 Hz) and alpha (7–18 Hz) power, again, at ISO troughs.

Schack et al. (2002) detected nested oscillations in human EEG during the delay period of a working memory task. They found strong instantaneous coherence between frontal theta activity (4–8 Hz) and the envelope of pre-frontal beta/gamma activity (20–40 Hz). Demiralp et al. (2007) have also found evidence for nested oscillation in EEG recordings made whilst subjects perceived known and unknown objects. The oscillations were in the theta (average 5.9 Hz) and gamma bands (average 40.1 Hz).

Mormann et al. (2005) analysed ECoG from the medial temporal lobes of epilepsy patients during a continuous word recognition paradigm. Words were presented visually and half of them were later repeated. They found PAC between 4 Hz theta oscillations and beta activity (10–20 Hz) in hippocampus and rhinal cortex. In addition, they found a modulation of gamma activity (40–50 Hz) by a 6 Hz theta cycle.

More recently, Canolty et al. (2006) recorded ECoG from a grid of electrodes over a left fronto-temporal region and found that high gamma (80–150 Hz) amplitude peaked at the trough of ongoing theta oscillations. This occurred during a broad range of cognitive and motor tasks, and the spatial coupling patterns showed a greater degree of similarity for more similar tasks.

At a recent conference, Osipova et al. (2007) presented PAC results on MEG data. During eyes-closed resting behaviour the power of gamma oscillations (30–80 Hz) was found to be coupled to the phase of alpha activity (8–13 Hz). Subjects with strong alpha also showed strong PAC and gamma activity was maximal at the peak of alpha oscillation. Osipova et al. (2007) hypothesize that the visual system is inhibited during most of the alpha cycle except at a certain phase (the peak).

The concept of nested oscillation is perhaps most fully developed in the work of Lakatos et al. (2005) who propose an oscillatory hierarchy underlying the EEG in which theta amplitude is driven by delta phase and gamma amplitude by theta phase. This proposal was supported by recordings from the primary auditory cortex of awake macaques where the frequency and phase of ongoing delta waves adapted to match stimulus properties, over a set of repeated trials.

Whilst there have therefore been many reports of nested oscillation in the literature, the methods used to test for them have been rather heterogeneous. One might reasonably ask: What is the best method for detecting nested oscillation? This is the question we address in this paper.

We compare three different methods that have been proposed in the literature and a fourth proposed in this paper. These are: (i) the modulation index method of Canolty et al. (2006), (ii) the phase-locking value approach of Vanhatalo et al. (2004) and Mormann et al. (2005), (iii) the envelope-to-signal correlation (ESC) method of Bruns and Eckhorn (2004) and (iv) a new general linear model (GLM) approach derived from ESC. We initially apply the methods to electrocorticographic (ECoG) data recorded during a working-memory task, and then to data from a simulated hippocampal interneuron network. Further simulations are then made to address the dependence of each measure on signal to noise level, coupling phase, epoch length, sample rate, signal nonstationarity, and multi-phasic coupling. Throughout the paper we use the terms nested oscillation and phase-amplitude coupling interchangeably.

## 2 Methods

All of the methods for detecting nested oscillation rely on bandpass filtering and the Hilbert transform. Firstly, a univariate time series x[n], sampled at times $t_{n}$ for n=1,…,N is bandpass filtered into the two frequency bands of interest. For the rest of the paper we assume that these are the θ (e.g. 4–8 Hz) and γ (e.g. 30–80 Hz) bands, but the approaches are of course generically applicable to any two frequency bands. Filtering then produces the signals $x_{\theta }\left[n\right]$ and $x_{\gamma }\left[n\right]$.

In this paper we designed finite impulse response (FIR) filters using Matlab’s signal processing toolbox function firls.m. To remove any phase distortion the filters were applied to the original time series in the forward and then the reverse direction (using Matlab’s function filtfilt.m).

The Hilbert transform (Papoulis, 1991)

(1) $$y\left[t\right]=H\left(x\left[t\right]\right)=\frac{1}{\pi }\int_{-\infty }^{\infty }\frac{x\left[\tau \right]}{t-\tau }d\tau$$

is then applied to each resulting time series. This transform converts a cosine wave into a sine wave and, more generally, delays the original signal by π/2 at each frequency. The instantaneous phase can then be computed using the relative ratios of the original and transformed signal. Application of the transform to our two filtered time series allows us to form the complex variables, or ‘analytic signals’

(2) $$\begin{array}{c} z_{\theta }\left[n\right]=x_{\theta }\left[n\right]+\text{i}y_{\theta }\left[n\right]=a_{\theta }\left[n\right]\exp\left(\text{i}\phi_{\theta }\left[n\right]\right)\\ z_{\gamma }\left[n\right]=x_{\gamma }\left[n\right]+\text{i}y_{\gamma }\left[n\right]=a_{\gamma }\left[n\right]\exp\left(\text{i}\phi_{\gamma }\left[n\right]\right) \end{array}$$

where $\phi_{\theta }\left[n\right]$ and $\phi_{\gamma }\left[n\right]$ are the instantaneous phases, and $a_{\theta }\left[n\right]$ and $a_{\gamma }\left[n\right]$ are the instantaneous amplitudes of the θ and γ oscillations. One can also apply a second Hilbert transform to the gamma amplitude and from it compute the phase of the gamma amplitude, $\phi_{a_{\gamma }}\left[n\right]$. We then remove the first and last $w_{\theta }$ samples from each phase and amplitude time series, where $w_{\theta }$ is the order of the filter for the theta band. This eliminates edge effects introduced by filtering and was found to improve the specificity of all PAC measures.

To obtain the results in this paper we used a filter order for the theta band, $w_{\theta }$, equivalent to two cycles of the central theta period. The order of the gamma band filter $w_{\gamma }$ was set to three cycles of the central gamma period.

**Fig. 1:**
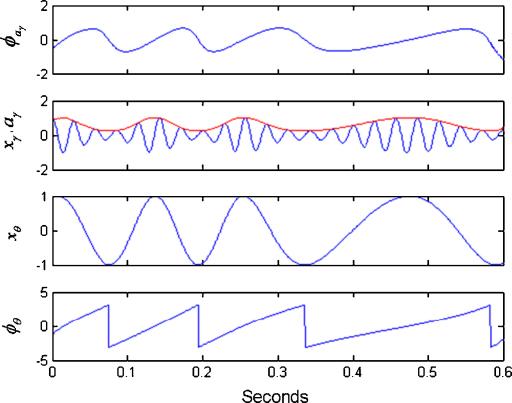
Instantaneous phase and amplitude. This figure shows the quantities necessary for computing the PAC measures. Firstly, the original signals are bandpass filtered to produce the time series $x_{\theta }$ and $x_{\gamma }$. Hilbert transforms are then applied from which one can estimate the gamma amplitude, $a_{\gamma }$ (shown in red) and the theta phase, $\phi_{\theta }$. One can then apply a Hilbert transform to the gamma amplitude to obtain the phase of the gamma amplitude, $\phi_{{\text{a}}_{\gamma }}$. (For interpretation of the references to color in the artwork, the reader is referred to the web version of the article.)

Fig. 1 illustrates the filtering and Hilbert transform steps required to produce the quantities necessary for computing the PAC measures. The above phases and amplitudes can also be estimated using a wavelet transform approach which produces similar results Le Van Quyen et al. (2001). The following subsections define three measures found in the literature to test for nested oscillation. The third subsection also describes the new GLM measure.

### 2.1 Modulation index

Canolty et al. (2006) define a ‘modulation index’ based on the complex variable

(3) $$z\left[n\right]=a_{\gamma }\left[n\right]\exp\left(\text{i}\phi_{\theta }\left[n\right]\right)$$

The absolute value of the mean vector is then given by

(4) $$M_{\text{raw}}=\left|\frac{1}{N}\overset{N}{\underset{n=1}{\sum }}z\left[n\right]\right|$$

where || denotes the absolute value. Assuming that $\phi_{\theta }\left[n\right]$ is uniformly distributed, any departure of the distribution of z[n] from radial symmetry will indicate a dependence of $a_{\gamma }\left[n\right]$ on $\phi_{\theta }\left[n\right]$. Therefore, a non-zero value of $M_{\text{raw}}$ will indicate PAC.

A significance value can be attached to $M_{\text{raw}}$ using a surrogate data approach. By introducing an arbitrary time lag between $\phi_{\theta }$ and $a_{\gamma }$ we can compute the surrogate complex variable $z_{\text{s}}\left[n\right]$. The mean of this over n=1,…,N is then given as $M_{s}$. This procedure is repeated to produce s=1,…,S surrogate values. From this surrogate data set we then compute the mean, μ and variance, $\sigma^{2}$, and compute a normalized modulation index

(5) $$M=\frac{M_{\text{raw}}-\mu }{\sigma }$$

and the *p*-value that corresponds to the standard Gaussian variate. This will only be an approximate *p*-value, however, as these samples are unlikely to be normally distributed. Our implementation of this method is based on the Matlab code provided in the supplementary material of Canolty et al. (2006). For the results in this paper, however, we used $M=M_{\text{raw}}$, rather than the normalized version in Eq. (5), since simulations showed it to have better statistical properties.

### 2.2 Phase-locking value

The phase-locking value (PLV), proposed by Lachaux et al. (1999) (see Tass et al., 1998 for a similar method), was developed to look at phase-locking between trials. Phase-locking factors have been previously used in neuroscience, for example, by Sinkkonen et al. (1995). Vanhatalo et al. (2004) and Mormann et al. (2005) have used the PLV in a different context; to look at PAC within a trial. In this context the two phases of interest are the phase of the theta oscillation, $\phi_{\theta }\left[n\right]$, and the phase of the γ amplitude, $\phi_{{\text{a}}_{\gamma }}\left[n\right]$. This latter phase is obtained with a second Hilbert transform from which we can produce the complex variable

(6) $$z_{a_{\gamma }}\left[n\right]=a_{a_{\gamma }}\left[n\right]\exp\left(\text{i}\phi_{{\text{a}}_{\gamma }}\left[n\right]\right)$$

The PLV is then defined as

(7) $$\text{PLV}=\left|\frac{1}{N}\overset{N}{\underset{n=1}{\sum }}\exp\left(\text{i}\left(\phi_{\theta }\left[n\right]-\phi_{{\text{a}}_{\gamma }}\left[n\right]\right)\right)\right|$$

where a value of unity indicates perfect phase-locking (in this context PAC), and a value of zero indicates no locking (no PAC).

As $a_{\gamma }\left[n\right]$ may contain non-theta-related components, these are first filtered out before extracting $\phi_{{\text{a}}_{\gamma }}\left[n\right]$, i.e. before the second Hilbert transform. In this paper, the filtering is implemented using the same bandpass filter used to obtain $x_{\theta }\left[n\right]$.

Phase-locking statistics (PLS) can then be derived using a standard surrogate data approach (Lachaux et al., 1999). This is implemented by randomly permuting $a_{\gamma }$ to produce surrogate time series $a_{\gamma }^{s}$ where s=1,…,S. PLV is then computed for each surrogate *s* resulting in a distribution of PLVs. The *p*-value of the original PLV can then be read off from this histogram.

### 2.3 Correlation and general linear model

Bruns and Eckhorn (2004) define an ESC measure as

(8) $$r_{\text{ESC}}={\text{Corr}}_{n}\left(x_{\theta }\left[n\right]\text{,}a_{\gamma }\left[n\right]\right)$$

In this paper we compute the correlation in the standard way

(9) $$\begin{array}{c} {\text{Corr}}_{n}\left(x\left[n\right]\text{,}y\left[n\right]\right)=\frac{1}{N}\frac{\overset{N}{\underset{n=1}{\sum }}\left(x\left[n\right]-\bar{x}\right)\left(y\left[n\right]-\bar{y}\right)}{\sigma_{x}\sigma_{y}}\\ \sigma_{x}^{2}=\frac{1}{N-1}\overset{N}{\underset{n=1}{\sum }}{\left(x\left[n\right]-\bar{x}\right)}^{2}\\ \sigma_{y}^{2}=\frac{1}{N-1}\overset{N}{\underset{n=1}{\sum }}{\left(y\left[n\right]-\bar{y}\right)}^{2}\\ \bar{x}=\frac{1}{N}\overset{N}{\underset{n=1}{\sum }}x\left[n\right]\\ \bar{y}=\frac{1}{N}\overset{N}{\underset{n=1}{\sum }}y\left[n\right] \end{array}$$

We stress that ESC is a very different measure than the correlation between $a_{\theta }\left[n\right]$ and $a_{\gamma }\left[n\right]$. This latter measure detects co-modulation of the amplitude envelopes and is referred to by Bruns and Eckhorn (2004) as amplitude envelope correlation (AEC). We denote it as

(10) $$r_{\text{AEC}}={\text{Corr}}_{n}\left(a_{\theta }\left[n\right]\text{,}a_{\gamma }\left[n\right]\right)$$

Similarly, a correlation between $a_{\theta }^{2}\left[n\right]$ and $a_{\gamma }^{2}\left[n\right]$ characterises correlations in power between the different frequency bands. This has been used before, by Friston (1996) for MEG data recorded during unilateral self-paced joystick movements, who observed that gamma frequency (35–40  Hz) oscillations in prefrontal cortex correlated with beta (18–20 Hz) frequency oscillations in parietal cortex.

The difference between the ESC and AEC measures is that, for ESC, the amplitude of the lower frequency oscillation is signed. As such, phase information is preserved. If for example $a_{\gamma }$ is largest when $x_{\theta }$ is most negative, and $a_{\gamma }$ is smallest when $x_{\theta }$ is most positive we have gamma peaks at theta troughs. This is PAC and will result in large negative ESC values.

We also note that large values of $r_{\text{AEC}}$ will not necessarily result in large values of $r_{\text{ESC}}$, particularly in cases of phase cancellation. Conversely, processes which generate large $r_{\text{ESC}}$ values will not necessarily produce large $r_{\text{AEC}}$ values. If PAC is intermittent, however, then one would expect a large overall $r_{\text{AEC}}$ and intermittently high $r_{\text{ESC}}$.

Because ESC can be confounded with amplitude co-modulations one could define an amplitude-normalized ESC measure as follows:

(11) $$r_{\text{NESC}}={\text{Corr}}_{n}\left(\cos\left(\phi_{\theta }\left[n\right]\right)\text{,}a_{\gamma }\left[n\right]\right)$$

This uses $\cos\left(\phi_{\theta }\left[n\right]\right)$ instead of $x_{\theta }\left[n\right]$ and so is invariant to amplitude modulations in $x_{\theta }\left[n\right]$, and hence to co-modulations in amplitude. The measure $r_{\text{NESC}}$ bears some similarity to the modulation index. If one takes the absolute operator inside the summation term in the definition of the modulation index in Eq. (4) and subtracts the mean from each term then one is left with $r_{\text{NESC}}$, up to a constant.

The above measure, $r_{\text{NESC}}$, however will still share a deficiency with ESC. This is an inability to detect coupling at 1/4 or 3/4 of a theta cycle (the so-called ‘null phases’ as, e.g. cos⁡((1/4)2π)=0) (Cohen, 2008). To ameliorate this, we propose a generalization of the above measure using the GLM framework used widely in neuroimaging (Friston et al., 2006). Gamma amplitude is modeled via a multiple regression

(12) $$a_{\gamma }=X\beta +e$$

where β are regression coefficients, *e* is additive Gaussian noise and the design matrix *X* is comprised of three columns, the first $\cos\left(\phi_{\theta }\left[n\right]\right)$, the second $\sin\left(\phi_{\theta }\left[n\right]\right)$ and the third is a column of 1s. One can then compute the proportion of variance explained by the model as

(13) $$r_{\text{GLM}}^{2}=\frac{\text{SS}\left(a_{\gamma }\right)-\text{SS}\left(e\right)}{\text{SS}\left(a_{\gamma }\right)}$$

where $\text{SS}\left(a_{\gamma }\right)$ is the sum of squares of the data and SS(e) is the sum of squares due to error. These can be computed in the usual way after model fitting (Christensen, 2002). As we shall see, the $r_{\text{GLM}}$ measure will be able to detect coupling at all theta phases, whereas this is not the case for $r_{\text{ESC}}$ or $r_{\text{NESC}}$.

When referring to the correlation measure in the rest of the paper, we mean the original ESC measure defined by Eq. (8), unless otherwise stated.

## 3 Results

### 3.1 ECoG data

This section describes application of the PAC measures to an ECoG data set. This data was recorded from a subject with intractable epilepsy who underwent temporary placement of an 8 × 8 grid of subdural electrodes placed over a fronto-temporal region including parts of sensorimotor cortex. The subject gave informed consent and the study was approved by the Institutional Review Board of the University of Washington School of Medicine. The electrode grid consisted of flat electrodes with an exposed diameter of 2.3-mm, inter-electrode distance of 1-cm and all electrodes were referred to an inactive electrode.

The subject took part in a working memory experiment in which he was presented with images of houses and indicated whether an image was the same as the previous one (a ‘target’) by closing their hand. For non-targets he was instructed to keep his hand relaxed and open. The subject was told there were a maximum of two of each image, therefore, after viewing a target knew the image did not have to be remembered for subsequent matching. Images were displayed for 600 ms and there was a 1600 ms delay before the next was presented. One hundred such images were presented and there were 20 ‘targets’, i.e. images that were the same as the previous one. There were therefore 80 non-target trials and 20 target trials.

The ECoG data was then processed as follows. The signals were amplified, bandpass filtered between 0.15 and 200 Hz and digitised at $f_{\text{s}}=1000$ Hz. Signals were later re-referenced to a common mean and notch filters applied to remove mains artefact at 60 and 120 Hz. We then filtered the data at each sensor into two bands: (i) theta (4–8 Hz) and (ii) the χ-band, using the bandpass filters described earlier. The χ-band is specified following the definition of a scale-free power law, has been found to correlate well with local cortical function (Miller et al., 2007, submitted for publication), and corresponds to frequencies between 76 and 200 Hz.

We then used the Hilbert transform as described earlier and for each trial, *j*, computed the Correlation measure, $r_{\text{ESC}}^{j}$, GLM measure, $r_{\text{GLM}}^{j}$, PLV measure, ${\text{PLV}}_{j}$, and modulation index, $m_{j}$. The PAC measures were then transformed into approximately Gaussian variates as follows. For the ESC and GLM measures we used Fisher’s *z*-transformation

(14) $$\begin{array}{c} z_{j}^{C}=\frac{1}{2}\log\left(\frac{1+r_{\text{ESC}}^{j}}{1-r_{\text{ESC}}^{j}}\right)\\ z_{j}^{G}=\frac{1}{2}\log\left(\frac{1+r_{\text{GLM}}^{j}}{1-r_{\text{GLM}}^{j}}\right) \end{array}$$

For the PLV, following Mormann et al. (2005), we used an arcsine transform

(15) $$z_{j}^{P}=\sin^{-1}\left(2{\text{PLV}}_{j}-1\right)$$

and for the modulation index we used a log-transform

(16) $$z_{j}^{M}=\log m_{j}$$

We also repeated the modulation index analysis without the log-transform and found little difference in results. We then compared the above *z*-scores for the target trials to the non-target trials using two-sample *t*-tests at each electrode. The *t*-tests for the correlation measures were applied to the absolute value of the *z*-scores. This is because we were not initially interested in whether condition differences were between negative or positive correlations. With this sign information removed, our significance scores are then commensurate with the GLM, PLV and modulation indices, that is, a positive PLV difference between conditions will correspond to a positive difference in correlation scores.

**Fig. 2:**
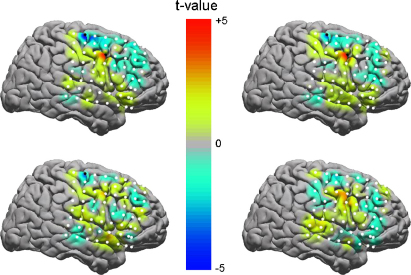
Task-related differences in nested oscillation. Maps of *t*-values for two-sample *t*-tests comparing PAC measures from ECoG data between target and non-target trials for ESC (top left), GLM (top right), PLV (bottom left) and modulation index (bottom right). In each image electrodes are numbered from bottom right (number 1) to top left (number 64). Electrodes marked with filled circles show significant differences between trial types. Both ESC and GLM indicate significant PAC differences at electrodes 63 and 45, and PLV at electrode 63. No significant differences were revealed by the modulation index. (For interpretation of the references to color in the artwork, the reader is referred to the web version of the article.)

The *p*-values associated with the *t*-tests were then corrected for multiple comparisons using a Bonferonni procedure. Only electrodes with p<0.05 (corrected) were deemed to contain significant differences. This corresponds to a *t*-score of 3.25. Fig. 2 shows the results for the ESC, GLM, PLV and modulation index measures. Both ESC and GLM indicate significant PAC differences at electrodes 63 and 45, and PLV at electrode 63. No significant differences were revealed by the modulation index.

**Fig. 3:**
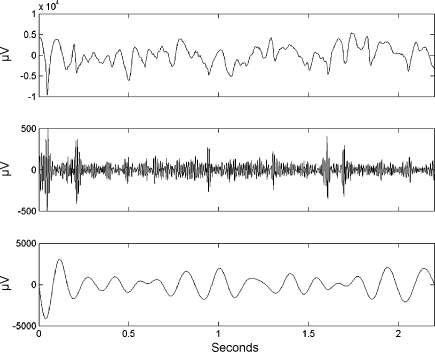
ECOG time series for non-target trial. Example non-target trial at electrode 63 showing the original time series (top), activity in the χ-band (middle) and activity in the theta band (bottom). The PAC measures are $r_{\text{ESC}}=-0.42$, $r_{\text{GLM}}=0.32$, PLV=0.57 and M=12.7.

**Fig. 4:**
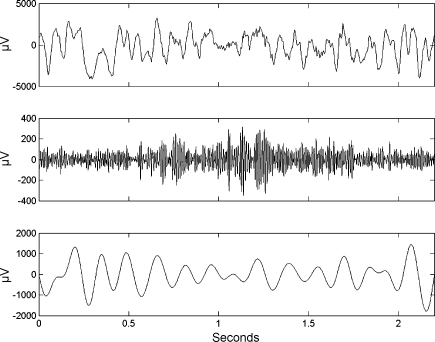
ECOG time series for target trial. Example target trial at electrode 63 showing the original time series (top), activity in the χ-band (middle) and activity in the theta band (bottom). The PAC measures are $r_{\text{ESC}}=0.02$, $r_{\text{GLM}}=0.06$, PLV=0.07 and M=6.8.

The first difference in the ESC measure, at electrode 63 (Talairach co-ordinates x=42, y=−15, z=59  mm) arises from no correlation for target trials but strongly negative correlation for non-target trials. Time series for each trial type are shown in Figs. 3 and 4. For the non-target trials one can see gamma bursts at theta troughs. For the target trials there is no such pattern. The PAC measures for the non-target trial are $r_{\text{ESC}}=-0.42$, $r_{\text{GLM}}=0.32$, PLV=0.57 and M=12.7. For the non-target trial the PAC measures are $r_{\text{ESC}}=0.02$, $r_{\text{GLM}}=0.06$, PLV=0.07 and M=6.8. Thus the modulation index does detect a difference over these two particular trials, but this effect did not reach statistical significance when all trials were considered.

For the second difference, at electrode 45 (Talairach co-ordinates x=52, y=5, z=39  mm), the pattern is reversed. That is, there is stronger coupling (at the theta trough) for target trials than for non-target trials. These between-trial differences may reflect hand movement rather than working memory processes, however, as subjects were instructed to close their hands for the target trials and keep them open for non-targets.

A concern with the ESC measure is that it is also sensitive to amplitude co-modulations. This is especially relevant for the ECoG data as amplitude co-modulations are clearly visible, for example, at the beginning of the non-target trial in Fig. 3. However, as the GLM measure also detects coupling changes we can be sure that there is significant phase-amplitude coupling in addition to any amplitude co-modulation.

### 3.2 Hippocampal interneuron network

**Fig. 5:**
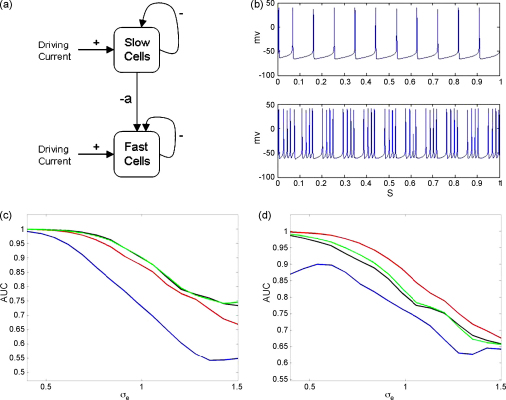
Hippocampal interneuron network. (a) Network model comprising a slow spiking population of GABA-A cells that causes a fast population of GABA-A cells to pause periodically and so generate a nested theta–gamma oscillation. All cells are driven with an externally applied current and the strength of the inhibition from the slow to the fast population is determined by the coupling parameter *a*. (b) Exemplar membrane potentials for fully synchronized slow spiking population (top) and fast spiking population (bottom) for a=0.6. The plots in the bottom row show the area under the curve (AUC) as a function of observation noise, $\sigma_{\text{e}}$, for the correlation measure (red), GLM measure (green), phase-locking value (black) and modulation index (blue) for (c) fully synchronized and (d) partially synchronized populations. (For interpretation of the references to color in the figure legend, the reader is referred to the web version of the article.)

This section describes application of the PAC measures to biophysically realistic simulated data. A network of Hippocampal interneurons was simulated following the approach described in White et al. (2000). Interneurons were partitioned into two populations, those with fast or slow ${\text{GABA}}_{A}$ synapses, such that the slow neurons generate a theta rhythm and the fast neurons a gamma rhythm. Neurons within each population are coupled together and, additionally, neurons in the slow population are coupled to those in the fast population, as shown in Fig. 5(a). This latter coupling causes the gamma rhythm to pause periodically every theta cycle and so generate a nested theta–gamma oscillation.

Details of the network parameters are described in Appendix A and broadly follow the simulations described in White et al. (2000). For the simulations in this paper we considered two scenarios. Firstly, we assumed perfect synchronization within each population. This allowed us to represent each population using the update equations for a single neuron. Secondly, we considered partial synchronization within each population and implemented this using the distributions of applied current described in Appendix A and by using five cells per population.

Fig. 5(b) shows the inhibition of the faster cells by the slower cells that leads to phase-amplitude coupling, which occurs for certain choices of the coupling parameter *a*. These time series were created as described in Appendix A. Simulated local field potentials (LFPs) were then created as the sum of the membrane potentials. The resulting signals were then downsampled, with appropriate anti-aliasing filters, to $f_{\text{s}}=512$  Hz, and normalized to zero-mean and unit variance. Different time series were created by different realizations of noise added on to the applied currents, as described in Appendix A. To these time series, we then added zero-mean Gaussian noise with variance $\sigma_{\text{e}}^{2}$.

In this and subsequent sections we quantify the performance of the various PAC measures using a receiver operating characteristic (ROC) approach (Swets, 1995). ROC curves plot the false positive versus the false negative rate as a decision threshold is varied. In this paper the decision thresholds were based on the PAC measures themselves rather than the corresponding *p*-values (for each measure, e.g. PLV, one can compute both the PLV and its associated *p*-value, as described above). ROC-curves based on the *p*-values produced almost identical results. For the simulations in this paper we computed ROC curves by first generating data from 100 PAC trials and 100 null trials, and varying the threshold over all empirically observed PAC values to compute the false positive and false negative rates. The AUC metric was then computed from these values (Swets, 1995) with a value of 0.5 indicating discrimination at the chance level and a value of 1 indicating perfect discrimination.

Fig. 5(c) and (d) shows how the AUC values fall off with increasing noise level for the different PAC measures. Each AUC value in these curves was derived by generating 100 trials of PAC data using coupling parameter a=0.6 and 100 trials of null data using a=0. The results were obtained using a ‘theta’ band from 5 to 20  Hz and a ‘gamma’ band from 50 to 130 Hz. The AUC curves have been smoothed for presentation purposes using a first-order bi-directional moving average filter. The modulation index performs significantly worse than the GLM, PLV or ESC measures for both fully and partially synchronized network dynamics.

The following subsections systematically examine the dependence of the PAC measures on signal to noise level, coupling phase, epoch length, sample rate, signal nonstationarity, and multi-phasic coupling. This examination takes place using a variety of synthetic signals which provide control over the type of coupling produced, e.g. preferred phase.

### 3.3 Sigmoidal coupling

We first generate a stationary theta oscillation

(17) $$x_{\theta }\left[n\right]=a_{\theta }\sin\left(2\pi f_{\theta }t\left[n\right]\right)$$

for samples n=1,…,N and then relate the amplitude of a gamma oscillation to it via the following sigmoidal nonlinearity

(18) $$a_{\gamma }\left[n\right]=\frac{k}{1+\exp\left(-c\left(x_{\theta }\left[n\right]-t_{c}\right)\right)}$$

**Fig. 6:**
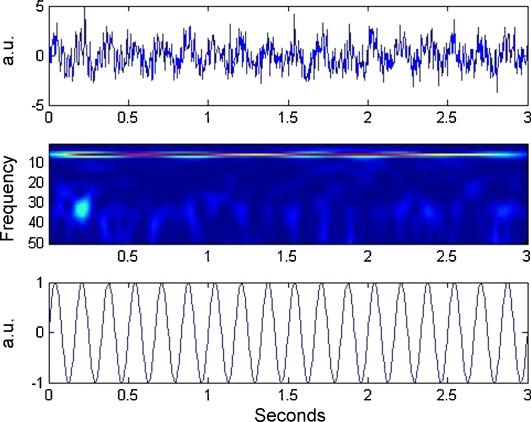
Sigmoidal coupling time series. A single trial of sigmoidal coupling data. The top plot shows a 3-s time series, the middle plot the corresponding spectrogram, and the bottom plot the theta oscillation used in generating the data. Note the gamma bursts at theta peaks. (For interpretation of the references to color in the figure legend, the reader is referred to the web version of the article.)

The gamma oscillation is given by

(19) $$x_{\gamma }\left[n\right]=a_{\gamma }\left[n\right]\sin\left(2\pi f_{\gamma }t\left[n\right]\right)$$

and an observed time series is then formed as follows:

(20) $$x\left[n\right]=x_{\theta }\left[n-n_{0}\right]+x_{\gamma }\left[n\right]+e\left[n\right]$$

where e[n] is zero mean Gaussian noise of standard deviation $\sigma_{\text{e}}$. The observed theta oscillation is delayed by a number of samples $n_{0}=\phi_{0}\left(f_{\text{s}}/f_{\theta }\right)$ which corresponds to a fraction, $\phi_{0}$, of a cycle. This allowed us to generate gamma bursts at different phases of the theta cycle. For the simulations in this section we use the following parameters. Theta amplitude and frequency are set to $a_{\theta }=1$, $f_{\theta }=6$ Hz, gamma frequency to $f_{\gamma }=35$ Hz and sigmoidal parameters to c=1, $t_{c}=0.95$. Fig. 6 shows an example time series of epoch length L=3  s and its spectrogram computed using a Morlet-wavelet time-frequency method. This data was generated using parameters k=2, $\sigma_{\text{e}}=1$, $n_{0}=0$ and $f_{\text{s}}=240$ Hz.

**Fig. 7:**
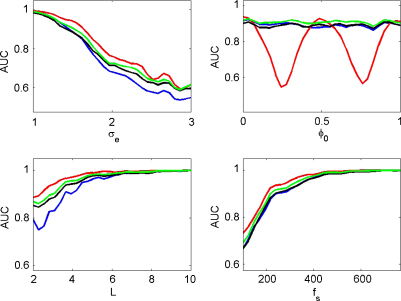
Sigmoidal coupling. The plots show the area under the curve (AUC) as a function of observation noise $\sigma_{\text{e}}$, coupling phase $\phi_{0}$, epoch length *L*, and sample rate $f_{\text{s}}$ for the correlation measure (red), GLM measure (green), phase-locking value (black) and modulation index (blue). (For interpretation of the references to color in the figure legend, the reader is referred to the web version of the article.)

Fig. 7 shows, for each of the measures, how the AUC varies as a function of observation noise, coupling phase, epoch length and sample rate. Each AUC value in these curves was derived by generating 100 trials of PAC data using gamma amplitude k=2 and 100 trials of null data using k=0. Parameters that were not varied for each plot were set to the default values of epoch length L=3  s, noise level $\sigma_{\text{e}}=1.5$, coupling phase $\phi_{0}=0$ and sample rate $f_{\text{s}}=256$ Hz. These AUC curves, and all others that follow, have been smoothed for presentation purposes using a first-order bi-directional moving average filter.

For low noise values, all methods are able to detect PAC almost perfectly. As the noise level increases the AUC falls, but there is a clear and consistent ordering, ESC having the highest AUC, followed by the GLM, PLV and then the modulation index. The simulation results show that the AUC for the ESC measure falls to chance levels at phases of 1/4 and 3/4 cycle, as expected, whereas the PLV and modulation indices are unaffected.

The lower plots in Fig. 7 show that all measures asymptote to the same AUC value of unity given sufficiently long epoch lengths or sufficiently high sampling rate. Before the asymptote, the ESC measure is consistently the most accurate, followed by GLM, PLV and the modulation index.

### 3.4 Von-Mises coupling

In this set of simulations the amplitude of gamma oscillations is given by

(21) $$a_{\gamma }\left[n\right]=\frac{c}{\exp\lambda }\exp\left(\lambda \cos\left(\phi_{\theta }\left[n\right]-2\pi \phi_{0}\right)\right)$$

where *c* controls the maximum gamma amplitude, $2\pi \phi_{0}$ the theta phase at which gamma is maximal and λ the phase concentration. In effect, λ controls the duty cycle, with large values causing gamma to be large only at phases very close to $2\pi \phi_{0}$, and a value of zero causing equally large gamma at all phases.

The form of the above equation was motivated by the Von-Mises probability density for phases (Papoulis, 1991), where the parameter λ is known as the ‘concentration parameter’ which, in turn, is analagous to the precision (inverse variance) parameter of a Gaussian density. We have replaced probability density with gamma amplitude, and chosen a normalization term such that the maximal value is *c*.

**Fig. 8:**
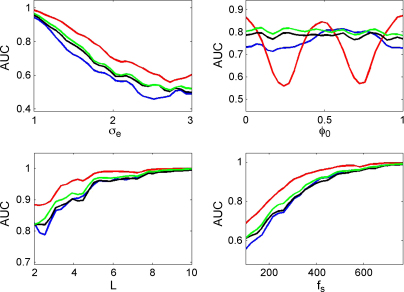
Von-Mises coupling. The plots show the area under the curve (AUC) as a function of observation noise $\sigma_{\text{e}}$, coupling phase $\phi_{0}$, epoch length *L*, and sample rate $f_{\text{s}}$ for the correlation measure (red), GLM measure (green), phase-locking value (black) and modulation index (blue). (For interpretation of the references to color in the figure legend, the reader is referred to the web version of the article.)

Fig. 8 shows, for each of the measures, how the AUC varies as a function of observation noise, coupling phase, epoch length and sample rate. Each AUC value in these curves was derived by generating 100 trials of PAC data using λ=1 and 100 trials of null data using λ=0. Parameters that were not varied for each plot were set to the default values of epoch length L=2.2  s, noise level $\sigma_{\text{e}}=1.5$, coupling phase $\phi_{0}=0$ and sample rate $f_{\text{s}}=256$ Hz.

In these simulations the ESC measure performs best over a wide range of noise levels, epoch lengths and sample rates, followed by GLM, PLV and the modulation index. Again, it shows its characteristic poor performance for coupling at null-phases. All measures asymptote to an AUC of unity for long epoch lengths and high sample rates.

### 3.5 Nonstationary theta

The simulations so far have used theta oscillations which are stationary throughout the examined epoch even though it is well known that electrophysiological signals are often nonstationary. For example, a theta oscillation from the ECoG data set shown in Fig. 3 shows clear amplitude and frequency modulation.

This section describes a set of simulations in which PAC data is generated as described in the previous section, but coupling is driven by a theta oscillation $x_{\theta }\left[n\right]$ that is derived from electrode 63 of the ECoG data described previously.

**Fig. 9:**
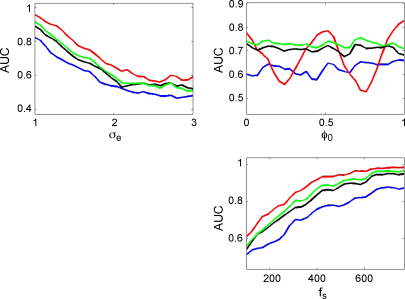
Nonstationary theta. The plots show the area under the curve (AUC) as a function of observation noise $\sigma_{\text{e}}$, coupling phase $\phi_{0}$ and sample rate $f_{\text{s}}$ for the correlation measure (red), GLM measure (green), phase-locking value (black) and modulation index (blue). (For interpretation of the references to color in the figure legend, the reader is referred to the web version of the article.)

In these simulations the epoch length is fixed to 2.2 s, as defined by the experiment. Fig. 9 shows, for each of the measures, how the AUC varies as a function of observation noise, coupling phase and sample rate. Each AUC value in these curves was derived by generating 100 trials of PAC data using λ=1 and 100 trials of null data using λ=0. Parameters that were not varied for each plot were set to the default values of noise level $\sigma_{\text{e}}=1.5$, coupling phase $\phi_{0}=0$ and sample rate $f_{\text{s}}=256$  Hz.

In these simulations the ESC measure performs best over a wide range of noise levels and sample rates, followed by GLM, PLV and the modulation index. Interestingly, the AUC asymptotes to unity before the experimental sample rate of $f_{\text{s}}=1000$ Hz is reached. The point at which this occurs is, however, a function of the noise level. For lower sample rates or higher noise levels the correlation measure produces the highest AUC scores.

**Fig. 10:**
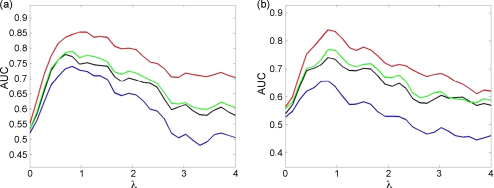
Effect of concentration parameter. The plots show the area under the curve (AUC) as a function of concentration parameter λ for: (a) Von-Mises coupling and (b) Von-Mises coupling with nonstationary theta for the correlation measure (red), GLM measure (green), phase-locking value (black) and modulation index (blue). (For interpretation of the references to color in the figure legend, the reader is referred to the web version of the article.)

Finally, Fig. 10 shows how AUC varies as a function of the concentration parameter, λ, for synthetic stationary theta epochs, and experimentally acquired nonstationary theta epochs. Each AUC value in these curves was derived by generating 100 trials of PAC data using the specified λ-value and 100 trials of null data using λ=0. Parameters that were not varied were set to the default values described above.

The ESC measure performs best in all cases, followed by GLM, PLV and the modulation index. The AUC peaks for a value of λ≈1. For low λ there is little or no PAC to be detected and for high λ the duration of the gamma bursts becomes too short to have sufficient SNR. The shape of these curves will also be determined by the length of the filter used to pass the gamma band (in this paper we have used filters of length three times the central gamma period).

Overall, Figs. 8–10 show lower AUC values for nonstationary data, indicating that activity that is nested in nonstationary oscillations is harder to detect than in stationary oscillations.

### 3.6 Biphasic coupling

This final section describes phase-amplitude coupled data generated according to a scenario similar to that described in Canolty et al. (2006). The aim is to produce a ‘synthetic signal [that] can be viewed as a very simple model of the activation and refractory period of a local neuronal population in a given cortical area’.

This is implemented using two sigmoidal nonlinearities

(22) $$\begin{array}{c} a_{\gamma }^{1}\left[n\right]=\frac{k_{1}}{1+\exp\left(-c_{1}\left(x_{\theta }\left[n\right]-t_{c}^{1}\right)\right)}\\ a_{\gamma }^{2}\left[n\right]=\frac{k_{2}}{1+\exp\left(-c_{2}\left(x_{\theta }\left[n\right]-t_{c}^{2}\right)\right)} \end{array}$$

with parameters chosen so that the first gamma amplitude increases at the theta trough, and the second at the theta peak. This was achieved using parameter values $c_{1}=-10$, $c_{2}=10$, $t_{c}^{1}=-0.95$, $t_{c}^{2}=0.95$. Each type of gamma burst was then switched on with probability 0.5 using binary switch variables $s_{1}$ and $s_{2}$. This resulted in gamma bursts at typically 50% of troughs and peaks and follows the suggestion of Canolty et al. (2006). The gamma oscillation is then given by

(23) $$x_{\gamma }\left[n\right]=\left(s_{1}a_{\gamma }^{1}\left[n\right]+s_{2}a_{\gamma }^{2}\left[n\right]+a_{\gamma }\right)\sin\left(2\pi f_{\gamma }t\right)$$

where the background gamma amplitude is $a_{\gamma }=2$. A time series was then formed as follows:

(24) $$x\left[n\right]=x_{\theta }\left[n-n_{0}\right]+x_{\gamma }\left[n\right]+e\left[n\right]$$

where $n_{0}=\phi_{0}\left(f_{\text{s}}/f_{\theta }\right)$ as before. We generated 100 trials of PAC data using gamma amplitudes $k_{1}=8$ and $k_{2}=4$ with noise level $\sigma_{\text{e}}=1$ and 100 trials of null data with identical parameters but gamma amplitudes of $k_{1}=k_{2}=0$. The amplitudes of the PAC data, $k_{1}$ and $k_{2}$, are deliberately set unequal so as to correspond to the proposed simulation described in the supplementary material of Canolty et al. (2006). AUC values were then computed as before.

**Fig. 11:**
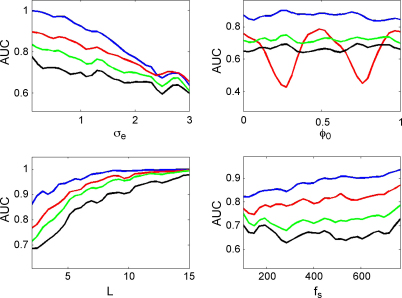
Biphasic coupling. The plots show the area under the curve (AUC) as a function of observation noise $\sigma_{\text{e}}$, coupling phase $\phi_{0}$, epoch length *L*, and sample rate $f_{\text{s}}$ for the correlation measure (red), GLM measure (green), phase-locking value (black) and modulation index (blue). (For interpretation of the references to color in the figure legend, the reader is referred to the web version of the article.)

Fig. 11 shows, for each of the measures, how the AUC varies as a function of observation noise, coupling phase, epoch length and sample rate. In these simulations, it is the modulation index which performs best over a wide range of noise levels, epoch lengths and sample rates. This confirms the assertion of Canolty et al. (2006), that the modulation index would be useful for detecting stochastic biphasic coupling. The PLV results can in principle be improved by looking for 2:1 instead of 1:1 phase locking, as described in Tass et al. (1998). However, no improvement was seen in these simulations. This is because at the simulated signal-to-noise ratios and after the relevant filtering and envelope extraction, only a change from high ($k_{1}=8$) to low ($k_{2}=4$) gamma activity could be extracted, and this occurs at theta frequency. Again, as expected, the ESC measure dips to chance values for increases of gamma at null theta phases.

## 4 Discussion

This paper has compared four different methods for detecting nested oscillation: (i) the modulation index of Canolty et al. (2006), (ii) the phase-locking value approach of Vanhatalo et al. (2004) and Mormann et al. (2005), (iii) the envelope-to-signal correlation (ESC) measure of Bruns and Eckhorn (2004) and (iv) a new measure based on a GLM. The GLM approach was motivated by improvements to the ESC measure. Firstly, by removing sensitivity to amplitude co-modulation and, secondly, by allowing coupling to be detected at all phases of the slower oscillation.

Application of the measures to an ECoG data set revealed significant differences in phase-amplitude coupling between target and non-target trials for electrodes placed over sensorimotor cortex. These were identified at two electrodes using the ESC and GLM measures and at one electrode using PLV. The modulation index also revealed differences but these did not reach statistical significance.

Application of the measures to biophysically realistic data from a simulated hippocampal interneuron network revealed similar findings. The ESC, GLM and PLV measures were better able to detect PAC than the modulation index over a broad range of signal to noise levels.

We then proceeded to characterise the dependence of the PAC measures on epoch length, sample rate, noise level, and coupling phase using two different simulation procedures. The first related gamma amplitude to the theta signal using a sigmoidal nonlinearity, and the second specified gamma amplitude using a nonlinearity motivated by the Von-Mises density. The conclusions, similar for both coupling methods, were that the ESC measure performed best. The caveat here, however, is that ESC is unable to detect PAC if the coupling occurs at 1/4 or 3/4 theta cycle (the so-called ‘null phases’). This is because the theta oscillation is equal to its mean level at these phases, therefore resulting in zero-correlation. This has also been remarked upon by Cohen (2008). The next best performing measure was GLM, closely followed by PLV.

A further set of simulations then addressed the issue of nonstationarity. To this end, theta oscillations from the ECoG data set were used and PAC data was generated using the Von-Mises method. The conclusions were again similar, that the ESC measure performed best, except for coupling at null phases. We also characterized the dependence of the PAC measures on the coupling duty cycle. This was implemented using Von-Mises coupling by varying the concentration parameter. The results showed that activity that is nested in nonstationary oscillations is harder to detect than in stationary oscillations, and that the ESC measure was best across a broad range of duty cycles. The next best performing measure was GLM, closely followed by PLV.

Finally, we addressed the issue of multi-phasic coupling. This refers to the possibility that gamma bursts may occur at multiple phases of the theta rhythm, but with different strengths. This issue was raised in the work of Canolty et al. (2006) who used it to motivate the definition of the modulation index. To this end, we generated biphasic PAC data using two sigmoidal nonlinearities. Our simulations showed the modulation index to be the best measure for detecting this sort of coupling.

Overall, our results suggest that ESC is the best measure for detecting PAC. However, the fact that ESC is insensitive to coupling at null phases presents a major drawback. In a recent rat electrophysiology study, for example, Jones and Wilson (2005) found that neurons in medial pre-frontal cortex spiked at approximately 1/4 of the hippocampal theta cycle. The ESC measure, applied to the relevant local fields, would be unable to detect this.

Our results on the ECoG, Hippocampal network, sigmoidal/Von-Mises coupling and nonstationary data sets show that GLM and PLV are better methods than the modulation index for short-epoch data. For this reason we recommend GLM, being slightly better than PLV, as the method of choice for data with short epochs (e.g. a few seconds) and the modulation index for longer epochs. The Jones and Wilson (2005) study, for example, used epochs of 2–2.5  s and our ECoG data epochs were 2.2 s. These are perhaps too short for the modulation index to be effective. In contrast, Canolty et al. (2006) applied the modulation index to very long data epochs, of duration 3–8 min.

A caveat to the above recommendation is that the modulation index is better able to detect biphasic coupling. But the biological relevance of such coupling remains to be established. On a more practical note perhaps our most useful finding is that one should use as long an epoch length and as high a sampling rate as is possible given the experimental constraints. This result is analagous to standard results in signal processing theory. For example, the accuracy with which the frequency of a single sinusoid in white noise can be estimated, has been shown to be inversely proportional to the epoch length and inversely proportional to the square root of the sampling rate (see Eq. (2.11) in Bretthorst, 1988).

We now turn to the question as to why ESC and PLV have better statistical properties than the modulation index, for short-epoch data. A possible reason, suggested to us by a reviewer of this paper, is related to the intrinsic dimensionality of each of the measures. If one looks inside the summation terms of each of the measures, then the ESC, defined in Eq. (8), concerns the product of two real numbers which is therefore one-dimensional, the PLV, defined in Eq. (7), is the product of conjugate complex exponentials which is therefore constrained to lie on the one-dimensional unit circle, whereas the modulation index, defined in Eq. (4), is intrinsically two-dimensional because it depends on the product of a real number and a complex exponential. As the detection of a one-dimensional quantity is more difficult when projected into two dimensions this may explain our observed results.

That intrinsic dimensionality plays a such a role is perhaps borne out by the following observation. We defined a simpler GLM approach using a single regressor, $\cos\left(\phi_{\theta }\left[n\right]\right)$ (due to the one-to-one mapping between regression and correlation this is equivalent to the NESC measure defined in Section 2). Like ESC it was unable to detect coupling at null phases but, also like ESC, it had higher AUC values at non-null phases (results not shown). We suggest that this is because there are fewer regression coefficients to estimate for the simpler model. If one knows at what phase the coupling will or will not occur then one can devise a better test. This is analagous to the well-known higher sensitivity of *t*-tests (that test for single effects) than *F*-tests (which test for linear combinations of multiple effects) in GLMs (Christensen, 2002).

We also note two possible extensions to the GLM test. Firstly, one could include extra regressors for higher order terms, e.g. $\cos\left(2\phi_{\theta }\left[n\right]\right)$ and $\sin\left(2\phi_{\theta }\left[n\right]\right)$, although this did not improve AUC scores for data sets in this paper. Secondly, for the analysis of multiple trial data, instead of using a Fisher-transformation of the correlation, $r_{\text{GLM}}$, one could use the regression coefficients themselves as summary statistics. This would then more closely follow the use of summary statistics for random effects analysis in neuroimaging (Friston et al., 2006).

Finally, the optimal method for detecting nested oscillation will depend upon the underlying biological mechanism. If one could construct a generative model that captures this mechanism, then by the Neyman–Pearson lemma, one could construct a likelihood-ratio test that has optimum detection performance. Recently, a modelling framework has been developed which allows one to fit differential equation models to neuroimaging data (Friston et al., 2003). A phenomenological version of this approach (Chen et al., 2008) has been applied to a class of M/EEG induced responses, which includes nested oscillation as a special case. Biophysical refinement of these models appears to be a fruitful direction for further research.

## Appendix A Neural network simulation

Neuron dynamics were modelled using single compartment Hodgkin–Huxley models as described in White et al. (1998). The membrane potential of the *i*th cell in the network evolves according to the following current balance equation:

(25) $$\frac{dV_{i}}{dt}=I_{i}-I_{\text{Na}}-I_{\text{K}}-I_{\text{L}}-I_{\text{s}}$$

where C=1 μF cm^−2^ and the currents are given by

(26) $$\begin{array}{c} I_{\text{Na}}=g_{\text{Na}}m_{\infty }^{3}h\left(V_{i}-V_{\text{Na}}\right)\\ I_{\text{K}}=g_{\text{K}}n^{4}\left(V_{i}-V_{k}\right)\\ I_{\text{L}}=g_{\text{L}}\left(V_{i}-V_{\text{L}}\right)\\ I_{\text{s}}=\overset{N}{\underset{j=1}{\sum }}A_{ij}\left(g_{\text{s}}/N\right)s_{j}\left(V_{i}-V_{\text{s}}\right) \end{array}$$

where $I_{\text{Na}}$ and $I_{\text{K}}$ are the Hodgkin–Huxley spike generating currents, $I_{\text{L}}$ is the leak current and $I_{\text{s}}$ is the synaptic current. The following conductances $g_{\text{Na}}=30$ mS cm^−2^, $g_{\text{K}}=20$ mS cm^−2^, $g_{\text{L}}=0.1$ mS cm^−2^ and equilibrium potentials $V_{\text{Na}}=45$ mV, $V_{\text{K}}=-80$ mV, $V_{\text{L}}=-60$ mV, $V_{\text{s}}=-75$ mV were used. We used connectivity values $A_{ij}=2$ for i,j in the same sub-network (i.e. fast or slow) and $A_{ij}=2a$ for the connections from the slow to the fast network. Nested oscillation is only observed for certain values of the coupling parameter *a* and this was varied as described in the main text so as to test the statistical detection properties of each PAC measure.

The inactivation variable *h* and the activation variable *n* are governed by the dynamics

(27) $$\begin{array}{c} \frac{dh}{dt}=\frac{h_{\infty }-h}{\tau_{h}}\\ \frac{dn}{dt}=\frac{n_{\infty }-n}{\tau_{n}} \end{array}$$

and the following voltage-dependent relations are used

(28) $$\begin{array}{c} h_{\infty }=\frac{1}{1+\exp\left(0.13\left(V+38\right)\right)}\\ n_{\infty }=\frac{1}{1+\exp\left(-0.0045\left(V+10\right)\right)}\\ m_{\infty }=\frac{1}{1+\exp\left(-0.08\left(V+26\right)\right)}\\ \tau_{h}=\frac{0.6}{1+\exp\left(-0.12\left(V+67\right)\right)}\\ \tau_{n}=0.5+\frac{2}{1+\exp\left(0.045\left(V-50\right)\right)} \end{array}$$

The gating variable $s_{j}$ was assumed to obey first-order kinetics of the form

(29) $$\frac{ds_{j}}{dt}=\frac{F\left(V_{j}\right)\left(1-s_{j}\right)}{\tau_{\text{rise}}^{j}}-\frac{s_{j}}{\tau_{\text{fall}}^{j}}$$

where, for fast cells $\tau_{\text{rise}}^{j}=1$ ms and $\tau_{\text{fall}}^{j}=9$ ms, for slow cells $\tau_{\text{rise}}^{j}=5$ ms and $\tau_{\text{fall}}^{j}=150$ ms, and

(30) $$F\left(V_{j}\right)=\frac{1}{1+\exp\left(-V_{j}\right)}$$

In the network simulation with a single cell per population we used $\tau_{\text{fall}}^{j}=100$ ms. The equations were integrated using a Runge–Kutta method with a step size of 0.5 ms. The applied currents $I_{i}$ were set to 2 μA plus a uniformly distributed random variable $e_{i}$ with maximum value $\sigma_{\text{app}}$. Larger values of $\sigma_{\text{app}}$ correspond to a more heterogeneous population, thus affecting the synchronization properties of the network. We used $\sigma_{\text{app}}=0.1$.
